# Treatment with PCSK9 monoclonal antibodies is associated with discontinuation of oral lipid lowering therapy

**DOI:** 10.1093/ehjqcco/qcae099

**Published:** 2024-11-19

**Authors:** Ingrid Engebretsen, Kristina Malene Ødegaard, Sigrun Halvorsen, Christoffer Bugge, Ivar Sønbø Kristiansen, Henrik Støvring, John Munkhaugen

**Affiliations:** Department of Behavioral Medicine, Faculty of Medicine, University of Oslo, Sognsvannsveien 9, 0372 Oslo, Norway; Oslo Economics, 0161 Oslo, Norway; Department of Medicine, Drammen Hospital, 3004 Drammen, Norway; Novartis Norway AS, 0484 Oslo, Norway; Department of Cardiology, Oslo University Hospital Ullevål, 0450 Oslo, Norway; Institute of Clinical Medicine, Faculty of Medicine, University of Oslo, 0450 Oslo, Norway; Oslo Economics, 0161 Oslo, Norway; Department of Health Management and Health Economics, University of Oslo, 0317 Oslo, Norway; Research Unit of General Practice, University of Southern Denmark, 5000 Odense, Denmark; Oslo Economics, 0161 Oslo, Norway; Steno Diabetes Center Aarhus, 8200 Aarhus, Denmark; Department of Biomedicine, Aarhus University, 8000 Aarhus, Denmark; Clinical Pharmacology, Pharmacy and Environmental Medicine, Department of Public Health, University of Southern Denmark, 5230 Odense, Denmark; Department of Behavioral Medicine, Faculty of Medicine, University of Oslo, Sognsvannsveien 9, 0372 Oslo, Norway; Department of Medicine, Drammen Hospital, 3004 Drammen, Norway

**Keywords:** Proprotein convertase subtilisin/kexin type 9 monoclonal antibodies, Adherence, Lipid lowering drugs, Drug adherence, The Norwegian Prescription Database

## Abstract

**Aims:**

Proprotein convertase subtilisin/kexin type 9 monoclonal antibodies (PCSK9 mAbs) are recommended for high-risk patients if the low-density lipoprotein cholesterol targets are not achieved with statins and ezetimibe. We studied persistence and adherence to (i) PCSK9 mAbs and (ii) statins and ezetimibe in a nationwide cohort of incident PCSK9 mAb users.

**Methods and results:**

Information on all PCSK9 mAb users ≤80 years from 2015 through 2023 were extracted from the Norwegian Drug Registry. Discontinuation was defined as a gap in treatment ≥180 days and ≥90 days. Adherence was measured as the proportion of days covered during the initial year of PCSK9 mAb therapy. We analysed adherence of statins and ezetimibe before and after PCSK9 mAb initiation. Of 4784 patients initiating PCSK9 mAbs, the median age was 63 years, 41% were female, 61% had atherosclerotic disease, and 34% had familial hypercholesterolaemia. Within 3 years after initiation, 17% experienced a PCSK9 mAb treatment gap exceeding 180 days. In the 12-month period preceding PCSK9 mAb initiation, 74% dispensed statins whereas 67% dispensed ezetimibe. These numbers were reduced to 35% for statins and 42% for ezetimibe during the 12-month period after PCSK9 mAb initiation. Atherosclerotic disease, using ≥3 statins previously, and older age were significantly associated with discontinuation of statins and ezetimibe.

**Conclusion:**

In this high-risk cohort of incident PCSK9 mAb users, more than 1 out of 2 stopped taking statin treatment whereas 40% discontinued ezetimibe. There is a major potential for improving adherence to oral LLD treatment following initiation of PCSK9 mAb.

## Graphical Abstract

**Graphical Abstract:**
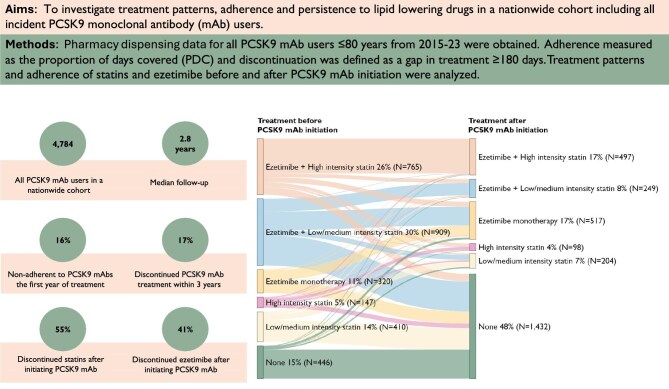

Key Learning Points What is already known

- Proprotein convertase subtilisin/kexin type 9 monoclonal antibodies (PCSK9 mAbs) are recommended for high-risk patients if the low-density lipoprotein cholesterol (LDL-C) targets are not achieved with statins and ezetimibe.
- There is limited knowledge on treatment patterns, adherence, and persistence to lipid lowering drugs among patients initiating PCSK9 mAbs, and nationwide data are lacking.

What this study adds

- Seventeen percent had treatment gaps exceeding 180 days and 28% had gaps exceeding 90 days within 3 years after PCSK9 mAb initiation.
- More than 50% stopped statin therapy after initiating PCSK9 mAb whereas 40% discontinued ezetimibe.
- Atherosclerotic disease, using ≥3 statins previously, and older age were significantly associated with discontinuation of statins and ezetimibe.

## Introduction

Healthy lifestyle and lipid lowering drug (LLD) treatment is crucial in halting the progression of atherosclerotic plaque and reducing the incidence of atherosclerotic cardiovascular disease (ASCVD) events.^1,2^ Statins are the cornerstone drug class reducing low-density lipoprotein cholesterol (LDL-C) by more than 50%,^1^ Ezetimibe provides a moderate additional effect on LDL-C.^3^ More recently, innovative drugs targeting the proprotein convertase subtilisin/kexin type 9 (PCSK9) receptors have emerged. PCSK9 monoclonal antibodies (PCSK9 mAbs) prevent the PCSK9 protein from degrading LDL receptors and reduce LDL-C by approximately 50% on top of usual care in randomized controlled trials.^4,5^ PCSK9 mAbs are strongly recommended as additional treatment to patients at very high risk when the LDL-C treatment targets are not achieved with statins and ezetimibe alone.^1^

PCSK9 mAbs have been available in Norway since 2015, but their accessibility has been governed by strict reimbursement rules. Prior to 2023, reimbursement was available after individual application for patients with familial hypercholesterolaemia (FH) and LDL-C >5.0 mmol/L or for patients with ASCVD and LDL-C >4.0 mmol/L. In 2023, the government announced the PCSK9 mAbs tender, which resulted in access to a broader patient population. Alirocumab was subsequently approved for general reimbursement for patients with FH and/or ASCVD with LDL-C above specific criteria despite ongoing treatment with highest tolerated statin dose and ezetimibe.^6^ For primary prevention in FH and ASCVD without additional risk enhancers, the LDL-C level must be >3.6 mmol/L, while for secondary prevention in FH or ASCVD with known risk enhancers (previous ASCVD and/or diabetes), the LDL-C level must be >2.6 mmol/L.

Despite availability, low patient copayment, and strong recommendations,^1,2^ underutilization and poor adherence to LLDs may explain why only few patients reach the recommended LDL-C levels in daily clinical practice.^7,8^ Previous studies have revealed that a considerable proportion of statin users in Norway have poor adherence whereas as much as 40% discontinue this treatment.^9^ There is limited knowledge on treatment patterns, adherence, and persistence to LLD among patients initiating PCSK9 mAbs. Previous studies investigating adherence to PCSK9 mAbs are constrained by short follow-up,^9,10^ small and selected patient samples^11^ or populations,^12,13^ lack of pharmacy registry data,^12–15^ and importantly, lack of information on concomitant oral LLDs.^14^ The aim of this study was to investigate treatment patterns, persistence, and adherence to LLD in a nationwide cohort including all incident PCSK9 mAb users.

## Methods

### Patient population

All pharmacies in Norway are mandated to send electronic data to the Norwegian Drug Registry (NDR), and the registry is therefore virtually complete.^16^ We obtained data from NDR for all pharmacy dispensed statins (ATC-code C10AA), ezetimibe (C10AX09), combinations of statin and ezetimibe (C10BA), PCSK9 mAbs (C10AX13 and C10AX14), and inclisiran (C10AX16) from 1st January 2004 through 31st August 2023. For patients with at least one such dispensing, we collected information on concomitant dispensed diabetes drugs (ATC-code A10), diuretics (C03), beta-blockers (C07), calcium channel blockers (C08), and renin-angiotensin inhibitors (C9).

We included all patients with at least one PCSK9 mAb dispensing in the analysis, and the patient's initial dispensing was defined as the index date. Because PCSK9 mAbs were introduced in Norway in 2015, all PCSK9 mAb users in the study were incident users. Patients were followed until death, reaching 81 years of age, or the end of data period (31st August 2023).

### Ethics

This project was evaluated by the Norwegian Directorate of eHealth (NDE) (NDE reference: H-72-E). NDE waived the requirement for written informed consent.

### Study variables

For each drug dispensing, we received information on patient characteristics (age, sex, region of residence), dispensing date, ATC-code and name of drug, number of defined daily doses (DDDs), pack size, strength, and reimbursement codes.

We defined the indication for PCSK9 mAb based on the reimbursement code (FH or ASCVD) registered at the patient's initial PCSK9 mAB dispensing. A physician can only indicate one reimbursement code on a prescription, and patients with both indications could not be identified. If a patient were dispensed at least two diabetes drugs at any point before index, we classified the patient as having diabetes mellitus.

### Outcomes

#### PCSK9 mAbs

##### Persistence (time on treatment).

We calculated persistence to PCSK9 mAb treatment as time from the index date until treatment discontinuation, defined as a treatment gap exceeding 180 or 90 days switching between different types of PCSK9 mAbs, and switching from PCSK9 mAbs to inclisiran, was not considered as discontinuation. Grace period of 90 and 180 days were applied to account for potential stock piling. Norwegian pharmacies are allowed by law to dispense drugs for maximum 3 months in advance. However, we also applied a more conservative grace period of 180 days to account for potential stock piling, and to reduce potential interruptions in treatment caused by e.g. hospitalizations. Where drug survival means that the patient continues use of a drug, we estimated drug survival for PCSK9 mAb treatment and hazard ratios for treatment discontinuation as additional indicators of persistence. To determine the duration of each PCSK9 mAb prescription, we used the DDD approach. This involves dividing the dispensed quantity by the DDD to estimate the prescription duration. For alirocumab, the recommended starting dose is 75 mg every other week, with an option to escalate to 150 mg every other week or 300 mg once a month if the LDL-C reduction target is above 60%. The DDD for alirocumab is 5.4 mg, but this solely reflects the standard starting dose of 75 mg every other week. Alirocumab is available in three concentrations: 75, 150, and 300 mg/mL. When dispensed at 150 mg/mL every other week or 300 mg/mL once a month, the DDD is considered as 10 mg. Therefore, we applied a DDD of 5.4 mg if the dispensed dose was 75 mg, and a DDD of 10 mg if the dispensed dose was 150 or 300. For evolocumab, the DDD of 10 mg was applied.

##### Adherence.

We evaluated adherence to PCSK9 mAbs by assessing the proportion of days covered (PDC) by drug intake during the initial year of PCSK9 mAb therapy. We determined PDC as the ratio of days covered with medication to the total number of days in the observation period (365 days). In analyses of adherence, we only included patients with at least 1 year follow-up after the index date. We calculated the proportion of patients with (i) PDC <0.5, (ii) PDC 0.5–0.79, and (iii) PDC ≥0.8. We classified patients as non-adherent if they had a PDC <0.8 during the first year of PCSK9 mAb treatment.

#### Statins and ezetimibe

##### Treatment patterns.

We analysed treatment patterns for statins and ezetimibe preceding PCSK9 mAb initiation by determining the proportion of patients receiving at least one prescription for either a high (rosuvastatin 20 mg/40 mg or atorvastatin 40 mg/80 mg)—or medium/low (all other drug classes and doses)–intensity statin and/or ezetimibe in the 12-month period preceding the index date. The treatment category to which a patient belonged was based on the last dispensed drug during that year. Similarly, we assessed the treatment patterns of statins and ezetimibe in the 12-months period following PCSK9 mAb initiation. Patients were considered to discontinue statin/ezetimibe treatment if they had a statin/ezetimibe dispensing in the year preceding PCSK9 mAb initiation, but not in the year following. We assessed the proportion dispensing ≥3 vs. <3 different statins prior to PCSK9 mAb initiation.

##### Adherence.

For patients with at least one statin dispensing during the first year prior to index, we calculated their adherence to statin treatment for each 1 interval ±3 years from the index date. Adherence to statin treatment was measured by PDC, assuming patients were prescribed one tablet per day. Patients who had not had their first ever statin prescription (since 2010) in the year of interest or were lost to follow-up before the year of interest, were excluded from these analyses.

#### Association between adherence to statins and persistence to PCSK9 mAbs

We investigated persistence to PCSK9 mAbs among patients who had low (PDC <0.5), medium (0.5 ≤ PDC < 0.8), and high (PDC <0.8) adherence to statins in the year preceding PCSK9 mAB initiation. Only patients with at least one statin dispensing in the year preceding PCSK9 mAb initiation were included in this analysis. Persistence to PCSK9 mAb was calculated as described above, with discontinuation defined as a treatment gap exceeding 180 days.

### Statistical analyses

Patient characteristics were reported by number and proportion (%) of patients. The number of patients in each region of residence was reported as the number of patients per 100 000 inhabitants in 2020.^17^

Persistence to PCSK9 mA (time on treatment) was presented in Kaplan-Meier plots, both for all patients and by patient subgroups. Comparisons between subgroups were performed using the log-rank test. We fitted proportional hazard models (Cox models) to identify predictors of PCSK9 mAb treatment gaps exceeding 180 days. We used age, sex, number of statins (≥3 vs. <3) dispensed prior to PCSK9 mAb initiation, region of residence, treatment indication, and year of initiation as covariates. We report hazard ratios and 95% confidence intervals. Statistical significance was assessed at the 5% level.

Adherence to LLDs was reported as the proportion of patients with a PDC <0.5, 0.5–0.79, and ≥0.8, as well as mean PDC with standard deviations. We compared adherence in subgroups by a two-sample z-test for difference in proportions.

## Results

### Patient characteristics

**Figure 1:**
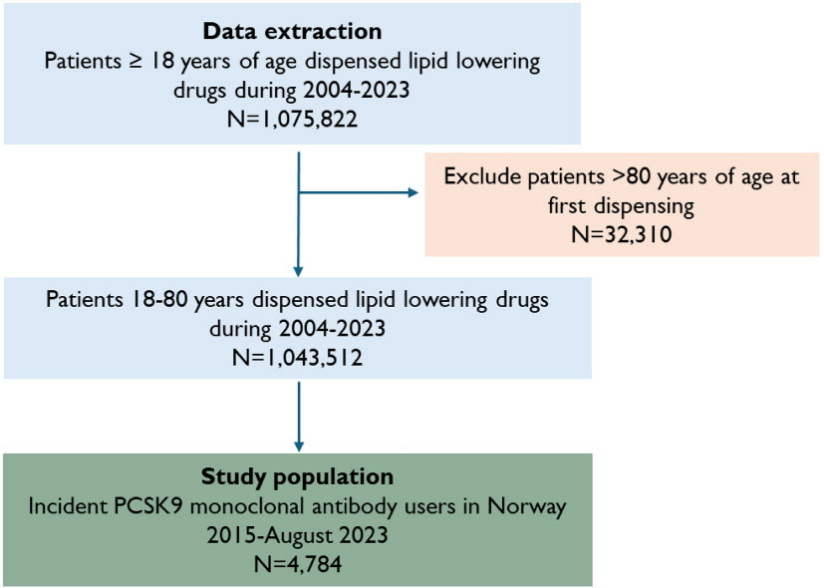
Flow chart.

The study flow is presented in *Figure 1*. In total, 4784 patients 18–80 years initiated PCSK9 mAb treatment from January 2015 through August 2023. The number of PCSK9 mAb users per year increased from 54 in 2015 to 4296 in 2023 (Supplementary material online, *Figure S1*). Median age at the date of the first PCSK9 mAb prescription was 63 (IQR: 16) years, and 41% (*N* = 1996) were female (*Table 1*). Sixty-one percent were prescribed PCSK9 mAb for ASCVD and 34% for FH while the rest had no reimbursement code stated on the prescription. In all, 46% had dispensed ≥3 different types of statins prior to PCSK9 mAb initiation.

**Table 1:** Patient characteristics at the date of the first PCSK9 monoclonal antibody prescription

	All patients
Number of patients	4784
Female (%)	1996 (40.8)
Median age (IQR) at index date	63 (16)
Age group at index date, *N* (%)	
<55	1207 (25.2)
55–63	1317 (27.5)
64–70	1188 (24.8)
71–80	1072 (22.4)
Region of residence, *N* per 100 000 inhabitants^a^	
North	119.7
Central	79.4
South-East	91.2
West	80.1
PCSK9 mAb indication, *N* (%)	
ASCVD	2916 (61.0)
FH	1626 (34.0)
None/unknown indication	242 (5.1)
Drug treatment for comorbid diseases, *N* (%)	
Diabetes mellitus	881 (18.4)
Insulins and analogues	288 (6.0)
Blood glucose lowering drugs, excl. insulins	798 (16.7)
Diuretics	1144 (23.9)
Beta blocking agents	2762 (57.7)
Calcium channel blockers	1568 (32.8)
Agents acting on the renin-angiotensin system	2765 (57.8)
Statin treatment prior to PCSK9 mAb initiation, *N* (%)	
<3 types of statins	2566 (53.6)
≥3 types of statins	2218 (46.4)

ASCVD, atherosclerotic cardiovascular disease; FH, familial hypercholesterolaemia; PCSK9 mAbs, proprotein convertase subtilisin/kexin type 9 monoclonal antibodies.

a Per 100 000 inhabitants in 2020.

### PCSK9 monoclonal antibodies

#### Persistence

Median follow-up was 2.0 (IQR: 3.5) years. The 1-year drug discontinuation (treatment gap ≥180 days) was 9% (95% CI: 8%, 10%), the 3-year drug discontinuation was 17% (95% CI:15%, 018%), whereas the 5-year drug discontinuation was 20% (95% CI: 19%, 21%). When applying a 90-day treatment gap as definition for treatment discontinuation, the 1-year drug discontinuation was 15% (95% CI: 14%, 16%), the 3-year drug discontinuation was 28% (95% CI: 26%, 30%), while the 5-year drug discontinuation was 35% (95% CI: 33%, 38%).

**Figure 2:**
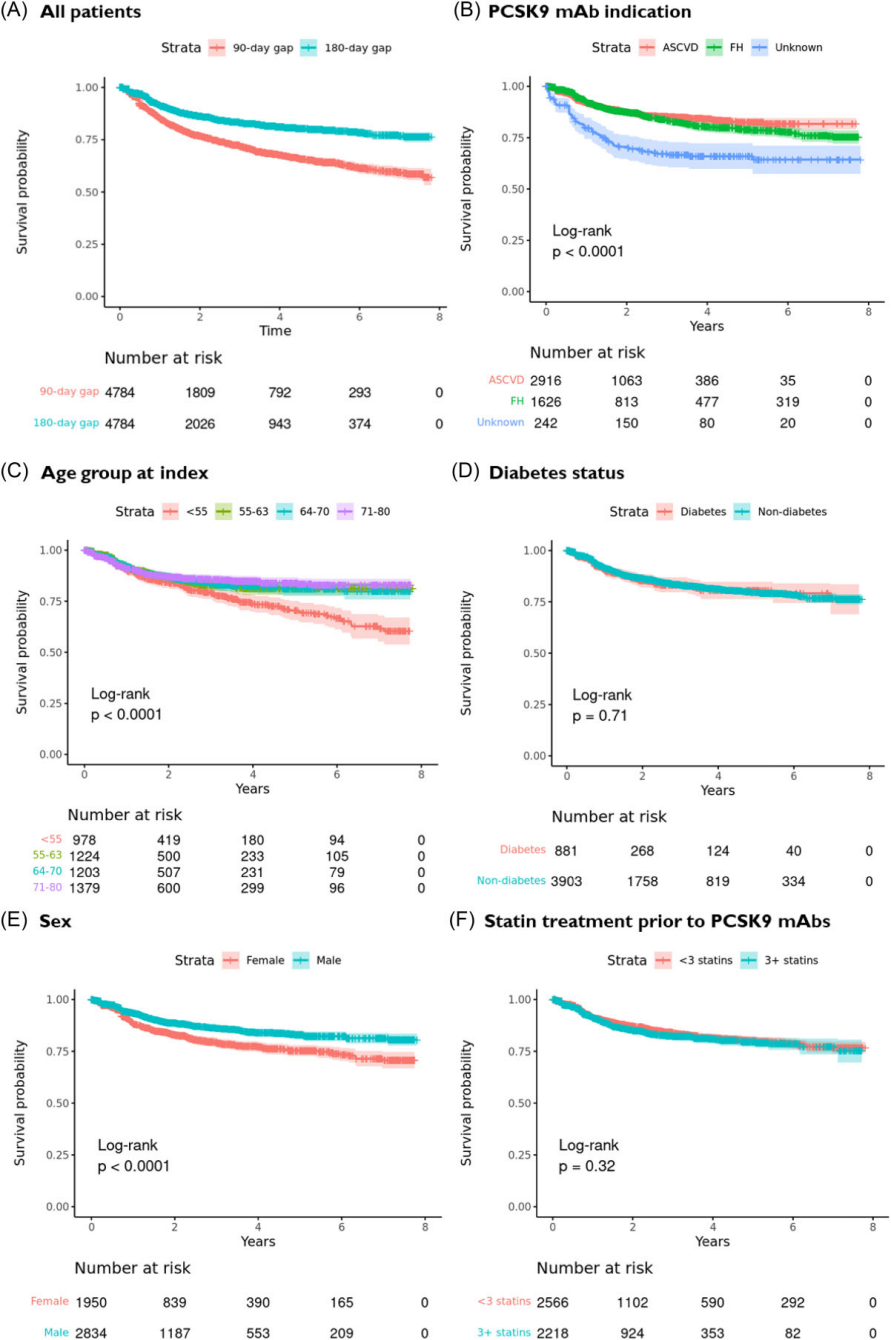
Persistence to PCSK9 mAbs (time until first 180-day treatment gap).

In bivariate analyses, discontinuation of PCSK9 mAb treatment was significantly higher among females compared with males, and among patients in the lowest age quartile (*Figure 2*). There were no significant differences in discontinuation according to treatment indication (ASCVD vs. FH), diabetes status or between patients who had dispensed ≥3 vs. <3 different statins prior to initiation of PCSK9 mAbs. Differences in persistence between patient subgroups were comparable when applying the 90-day and 180-day cut-offs (Supplementary material online, *Figure S2*). In multivariable analyses, similar results were observed when adjusting for sex, age, year of initiation, PCSK9 mAb indication, and region of residence (Supplementary material online, *Table S1*).

#### Adherence

There were 2997 patients with ≥1 year follow-up after index prescription (median age: 62 years, 43% female). During the first year of PCSK9 mAb treatment, mean PDC was 0.91 [SD: 0.21] and 16% had a PDC <0.8 (*Table 2*). Non-adherence to PCSK9 mAb was significantly higher in females compared with males (18% for females vs. 15% for males, *P* = 0.01), and in the lowest (18% in the lowest quartile vs. 13% in third quartile, *P* < 0.001) and highest (18% in the highest quartile vs. 13% in third quartile, *P* = 0.02) age quartiles compared with the third age quartile. There were no differences in adherence between patients with ASCVD and FH (15% for ASCVD vs. 14% for FH, *P* = 0.70), diabetes vs. no diabetes (18% in diabetes vs. 16% in non-diabetes, *P* = 0.23), or between patients who had dispensed ≥3 vs. <3 different types of statins prior to initiation of PCSK9 mAbs (16% for ≥3 statins vs. 16% for <3 statins, *P* = 0.51).

**Table 2:** Adherence to PCSK9 monoclonal antibody treatment

		PDC during first year of PCSK9 mAb treatment			
	Number of patients	Mean [SD]	PDC <0.5 *N* (%)	PDC 0.5–0.79 *N* (%)	PDC ≥0.8 *N* (%)
All patients	2997	0.91 [0.21]	270 (9.0)	208 (6.9)	2519 (84.1)
With diabetes mellitus	433	0.89 [0.23]	49 (11.3)	29 (6.7)	355 (82.0)
Without diabetes mellitus	2564	0.91 [0.21]	221 (8.6)	179 (7.0)	2164 (84.4)
Male	1709	0.91 [0.20]	127 (7.4)	120 (7.0)	1462 (85.5)
Female	1288	0.89 [0.23]	143 (11.1)	88 (6.8)	1057 (82.1)
PCSK9i indication					
ASCVD	1569	0.91 [0.21]	134 (8.5)	95 (6.1)	1340 (85.4)
FH	1200	0.92 [0.19]	86 (7.2)	82 (6.8)	1038 (86.5)
None/unknown indication	228	0.77 [0.32]	50 (21.9)	31 (13.6)	147 (64.5)
Age quartiles at PCSK9 mAb initiation					
<54 years	769	0.89 [0.21]	75 (9.8)	66 (8.6)	628 (81.7)
54–62 years	808	0.92 [0.20]	62 (7.7)	56 (6.9)	690 (85.4)
63–69 years	751	0.92 [0.21]	61 (8.1)	39 (5.2)	651 (86.7)
70–80 years	669	0.89 [0.24]	72 (10.8)	47 (7.0)	550 (82.2)
Statin treatment prior to PCSK9 mAb initiation					
<3 types of statins	1542	0.90 [0.22]	143 (9.3)	110 (7.1)	1289 (83.6)
≥3 types of statins	1455	0.91 [0.21]	127 (8.7)	98 (6.7)	1230 (84.5)

PDC, proportion of days covered; ASCVD, atherosclerotic cardiovascular disease; FH, familial hypercholesterolaemia; PCSK9 mAb, proprotein convertase subtilisin/kexin type 9 monoclonal antibodies.

### Statins and ezetimibe

#### Treatment patterns

**Figure 3:**
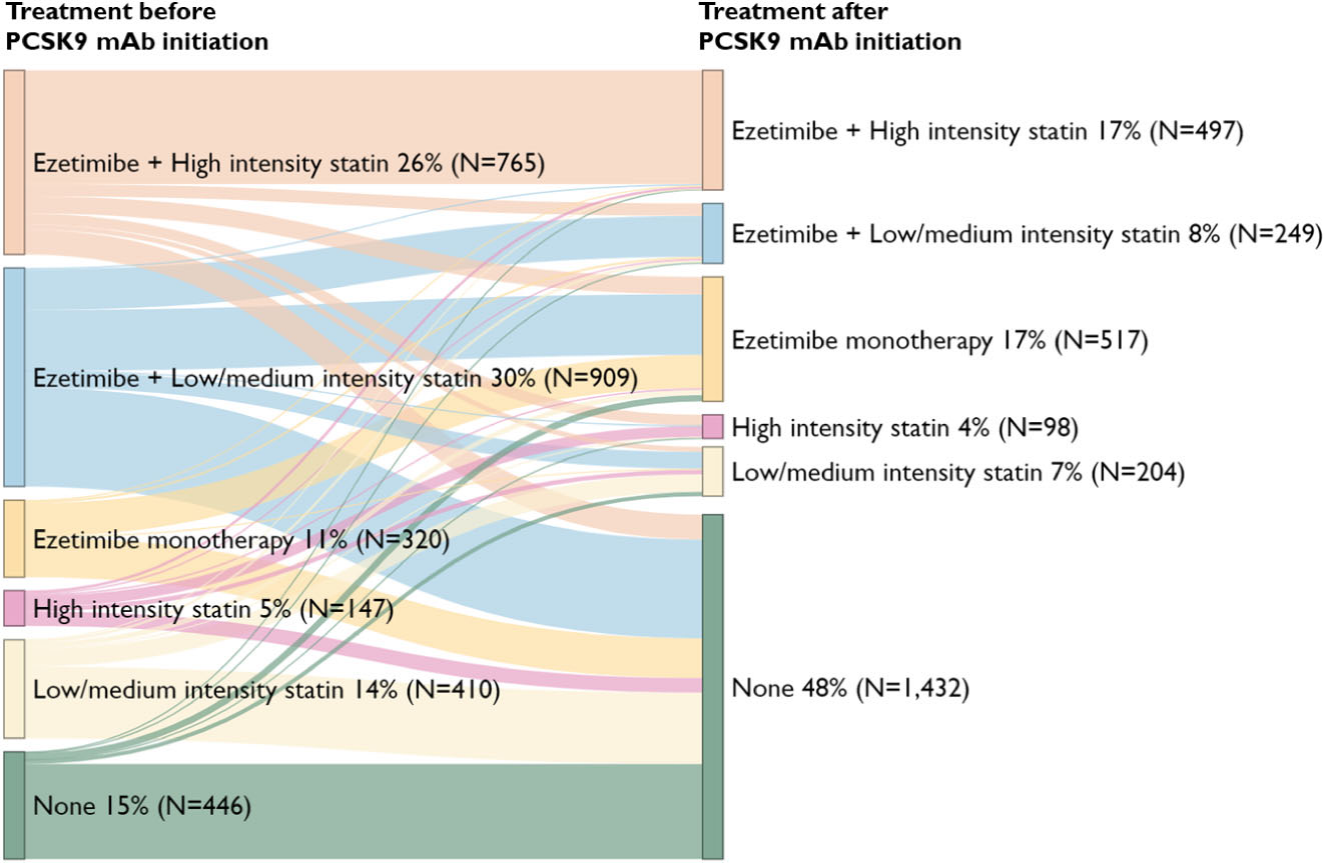
Sankey diagram showing the proportion of patients on different types of lipid-lowering drugs (statin and ezetimibe) in the year before and the year after initiation of PCSK9 mAb. Lines between bars show flows from one treatment group to another show flows from one treatment group to another.

In the 12-month period preceding PCSK9 mAb initiation, 2231 out of 2997 (74%) had at least one statin dispensed (*Figure 3*). Of these, 41% (*N* = 912) used a high-intensity statin whereas 59% (*N* = 1319) used a medium or low-intensity statin. In the 12-month period following PCSK9 mAb initiation, the proportion with at least one statin dispensing was reduced to 35% (*N* = 1048).

#### Persistence and adherence

In total, 55% (*N* = 1224) of patients discontinued statin treatment after PCSK9 mAb initiation, and 41% (*N* = 819) discontinued ezetimibe (*Table 3*) whereas 514 patients discontinued both statins and ezetimibe. Patients with ASCVD had significantly higher discontinuation rates compared to those with FH for both statins (71% for ASCVD, 36% for FH, *P* < 0.001) and ezetimibe (51% for ASCVD, 29% for FH, *P* < 0.001). Patients in the highest age quartile also had significantly higher discontinuation rates of statins (69% in highest vs. 42% in lowest, *P* < 0.001) and ezetimibe (50% in highest vs. 35% for lowest, *P* < 0.001) compared with those in the lowest age quartile, whereas no significant gender differences were observed for statins (55% for females, 55% for males, *P* = 0.952) or ezetimibe (41% for female, 41% for males, *P* = 0.813). Patients who had dispensed ≥3 different statins prior to PCSK9 mAb initiation had significantly higher discontinuation rate of statins (74% vs. 38%, *P* < 0.001) and of ezetimibe (54% vs. 30%, *P* < 0.001) compared with those dispensing <3 different statins.

**Table 3:** Statin and ezetimibe dispensing in clinical subgroups the year preceding and the year following PCSK9 monoclonal antibody initiation

	Statin dispensing in the year preceding and following PCSK9 mAb initiation *N* (%)			Ezetimibe dispensing in the year preceding and following PCSK9 mAb initiation *N* (%)		
	Year preceding	Year following	Discontinued statins	Year preceding	Year following	Discontinued ezetimibe
All patients (*N* = 2997)	2231 (74.4)	1048 (35.0)	1224 (54.9)	1994 (66.5)	1263 (42.1)	819 (41.1)
With diabetes mellitus (*N* = 433)	296 (68.4)	117 (27.0)	188 (63.5)	277 (64.0)	174 (40.2)	117 (42.2)
Without diabetes mellitus (*N* = 2564)	1935 (75.5)	931 (36.3)	1036 (53.5)	1717 (67.0)	1089 (42.5)	702 (40.9)
Male (*N* = 1709)	1320 (77.2)	616 (36.0)	723 (54.8)	1188 (69.5)	747 (43.7)	491 (41.3)
Female (*N* = 1288)	911 (70.7)	432 (33.5)	501 (55.0)	806 (62.6)	516 (40.1)	328 (40.7)
PCSK9 mAb indication						
ASCVD (*N* = 1569)	1127 (71.8)	349 (22.2)	799 (70.9)	1007 (64.2)	533 (34.0)	511 (50.7)
FH (*N* = 1200)	927 (77.3)	614 (51.2)	927 (35.8)	838 (69.8)	629 (52.4)	246 (29.4)
None/unknown indication (*N* = 288)	177 (77.6)	85 (37.2)	93 (52.5)	149 (65.4)	101 (44.3)	62 (41.6)
Age quartiles at PCSK9 mAb initiation						
<54 years (*N* = 769)	609 (79.2)	364 (47.3)	254 (41.7)	556 (72.3)	393 (38.0)	195 (35.1)
54–62 years (*N* = 808)	632 (78.2)	303 (37.5)	337 (53.3)	551 (68.2)	355 (43.9)	216 (39.2)
63–69 years (*N* = 751)	543 (72.3)	226 (30.1)	324 (59.7)	484 (64.5)	297 (39.6)	206 (42.6)
70–80 years (*N* = 669)	447 (66.8)	155 (23.2)	309 (69.1)	403 (60.2)	218 (32.6)	202 (50.1)
Statin treatment prior to PCSK9 mAb initiation						
<3 different statins (*N* = 1542)	1160 (75.2)	746 (48.4)	436 (37.6)	1081 (70.1)	799 (51.8)	328 (30.3)
≥3 different statins (*N* = 1455)	1071 (73.6)	302 (20.8)	1071 (73.6)	913 (62.8)	464 (31.9)	491 (53.8)

Discontinuation proportion refers to the proportion of patients who had a statin/ezetimibe dispensing in the year preceding PCSK9 mAb initiation, but not in the year following.

ASCVD, atherosclerotic cardiovascular disease; FH, familial hypercholesterolaemia; PCSK9 mAb, proprotein convertase subtilisin/kexin type 9 monoclonal antibody.

**Figure 4:**
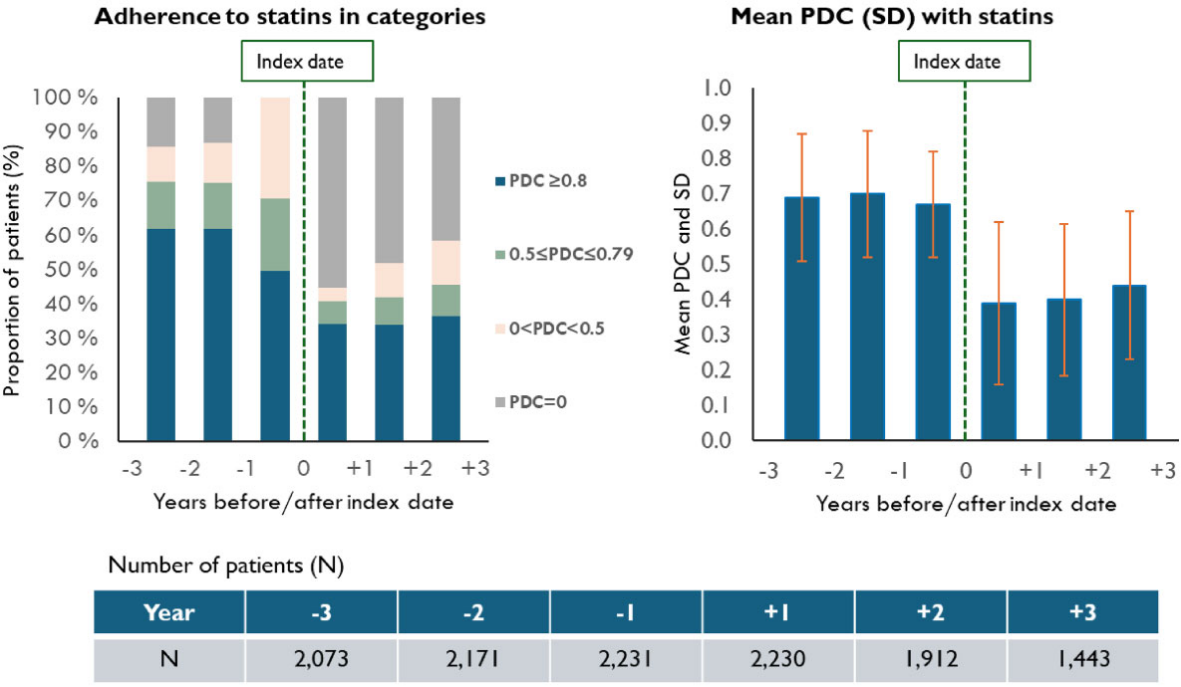
Adherence to statins before and after index date for patients with at least one statin dispensing during the year preceding PCSK9 mAb initiation (*N* = 2231). PDC, proportion of days covered; SD, standard deviation.

During the 12-month period preceding PCSK9 mAb initiation, 50% of those who had at least one statin dispensed had a PDC with statins ≥0.8, 21% had a PDC between 0.5 and 0.79, and 29% had a PDC <0.5. During the 12-months following PCSK9 mAb initiation, 34% of these patients had a PDC with statins ≥0.8, 7% had a PDC between 0.5 and 0.79, 4% had a 0 < PDC < 0.5, and 55% had a PDC = 0 (*Figure 4*).

#### Association between adherence to statins and persistence to PCSK9 mAbs

**Figure 5:**
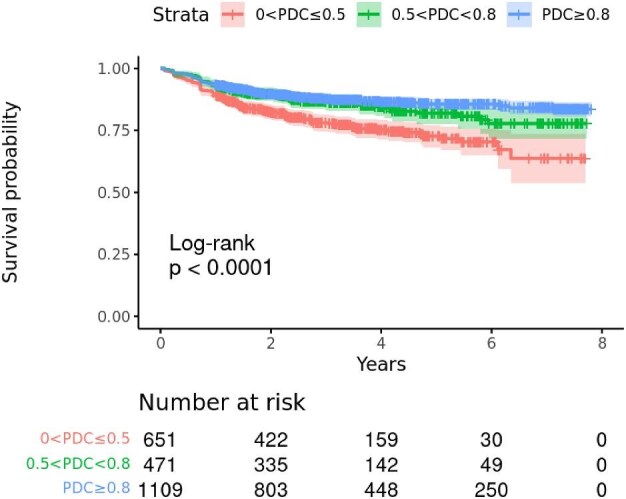
Association between adherence to statins and persistence to PCSK9 mAbs.

Persistence to PCSK9 mAbs was lower throughout follow-up for patients with lower adherence to statins (*Figure 5*). The 3-year drug discontinuation of PCSK9 mAb was 22% (95% CI: 19–25%) in patients a PDC of statins <0.5, 13% (95% CI: 11–17%) in those with 0.5 ≤ PDC < 0.8, and 12% (95% CI: 10–14%) in those with PDC ≥0.8.

## Discussion

In this nationwide cohort study of all Norwegian patients <80 years initiating PCSK9 mAb from 2015 through August 2023, we observed that almost one in five high-risk patients had a treatment gap with PCSK9 mAbs treatment exceeding 180 days, while almost one in three had a treatment gap exceeding 90 days. Poor adherence was most common among females and the youngest patients. It is particularly concerning that more than 1 out of 2 patients discontinue strongly recommended statin therapy after initiation of a PCSK9 mAb.

### Adherence and persistence to oral lipid lowering therapy

Ongoing treatment with the highest tolerated statin dose and ezetimibe is a criterion for reimbursement of PCSK9 mAbs in Norway. Only three in four patients used statins in the year preceding PCSK9 mAb initiation whereas only one in three used a high-intensity statin regime. The high discontinuation rate of statins and ezetimibe following initiation of PCSK9 mAbs is indeed surprising in a high-risk population with FH or established ASCVD or FH.

Our study revealed a remarkably higher discontinuation rate of statins and ezetimibe among patients with ASCVD (71%), compared to those with FH (36%). There are several possible explanations for this observation. First, most FH patients in Norway are closely followed-up at lipid clinics and are recommended to visit their general practitioner annually to measure their lipid levels. In contrast, there are no national recommendations for follow-up of chronic ASCVD patients, and a recent nationwide study revealed that only 14% of post-myocardial infarction patients attended a cardiac rehabilitation program.^18^ As lipid clinic physicians are highly experienced in the management of dyslipidaemias, FH patients may have received more information on the importance of ongoing treatment with oral LLD, compared with ASCVD patients. Second, patients with FH are more likely to have family members with early onset ASCVD due to strong heredity, potentially further increasing their health literacy and urge to comply with preventive recommendations. Third, FH patients usually have much higher levels of LDL-C than patients with ASCVD requiring multiple LLDs to reach the target. Lastly, it is important to consider the possibility of a selection effect within the group of ASCVD patients who receive PCSK9 mAbs. To qualify for reimbursement of PCSK9 mAbs in Norway, the LDL-C level must be above 3.6 mmol/L for most patients. While patients with FH mainly were at a high risk of ASCVD events due to elevated LDL-C levels, the causal framework is more complex for patients with established atherosclerosis. In addition to elevated LDL-C levels, poor lifestyle behaviour including smoking, low physical activity and, importantly, non-adherence to medication are important risk factors for ASCVD.^19^ Patients with ASCVD non-adherent to statins will not experience the LDL-C reduction produced by statins, leading to persistently elevated LDL-C levels. Consequently, these patients may potentially be more likely to meet the eligibility criteria for PCSK9 mAbs. Thus, it is possible that the sample was biased towards individuals hesitant to take statins and ezetimibe, which may explain the high discontinuation rates of oral LLDs.

The low proportion of patients on a high-intensity statin regimen and high discontinuation rate may be explained by subjective (self-perceived) side effects or intolerance.^20^ Patients who experience side effects are recommended to switch to another type of statin or lower doses.^1^ At the time of PCSK9 mAb initiation, almost half of the patients in our cohort had used three or more different types of statins. Discontinuation of statins after PCSK9 mAb initiation was significantly more prevalent in these patients (74%) compared with those previously dispensing less than three statins (38%). This may indicate that a considerable proportion of patients experience side effects. Previous observational studies have reported prevalence of statin side effects ranging from 9 to 12%.^21,22^ However, recent data from randomized crossover trials, including our own study among patients with self-perceived statin side effects demonstrate similar intensity of muscle symptoms during treatment periods with high-intensity atorvastatin (i.e. 40 mg) and placebo.^23^ Importantly, more than 9 out of 10 these trial participants tolerated statin treatment after careful information and re-introduction.^24^ Efforts to further strengthen patient information and education on this topic seems crucial. On the other hand, patients who had dispensed three or more different types of statin also had the highest discontinuation rate of ezetimibe even though this is regarded as a well-tolerated drug.^1^ Further, low adherence to statins before initiation of PCSK9 mAbs was significantly associated with higher discontinuation rates of PCSK9 mAbs.

Clinical inertia and/or misinformation in the communication between the physician and the patient are therefore also potential explanations for the poor adherence and persistence to LLDs.^25^ Although not recommended,^1,2^ physicians may have recommended discontinuation of oral LLD because of subjective side effects or because LDL-C fell well below the LDL-C targets after PCSK9 mAb initiation.

Studies investigating treatment patterns for statins and ezetimibe among PCSK9 mAbs users are scarce. A small US study found significantly lower statin adherence among PCSK9 mAb users (*N* = 178) compared with non-PCSK9 mAb users, and 28% of the PCSK9 mAb users discontinued their statins.^11^ In line with our results, a recent study from Sweden investigating adherence to evolocumab reported that only two in three patients used oral lipid lowering treatment at the time of PCSK9 mAb initiation whereas 48% discontinued statin treatment during the first year after initiation.^26^ The present study extends these findings to a nationwide cohort with data in treatment indication and in different patient subgroups.

### Adherence and persistence to PCSK9 mAbs

Continuous treatment with PCSK9 mAbs has proven to result in long-term reductions in LDL cholesterol levels.^27^ Even though 1-year adherence to PCSK9 mAb treatment was quite high with a mean PDC of 0.9, 16% had a PDC below 0.8 during the first year of PCSK9 mAb treatment. Further, 15% had a treatment gap exceeding 90 days during the first year of PCSK9 mAb treatment. This indicates that one in six patients had poor adherence and persistence with PCSK9 mAb treatment during the first year of treatment. As expected, drug survival further decreased over the 5-year period.

The risk of discontinuing PCSK9 mAb treatment was 70% higher for females compared with males even after adjustment for age, PCSK9 mAb indication, year of treatment initiation and region of residence. Further, adherence and persistence were significantly worse among the youngest patients. This is particularly worrisome as these would benefit the most from aggressive lipid lowering treatment in a lifelong perspective. Lower adherence among females and younger patients has previously also been documented for statin therapy.^9^ Contrary to oral LLDs, we did not observe differences in adherence or persistence to PCSK9 mAbs among patients with ASCVD compared to patients with FH. Patients with no reimbursement code stated in the prescription had significantly higher long-term discontinuation rate compared with others. The latter is not surprising as no reimbursement code means that the patient must pay for the treatment themselves.

Our findings extend the results from recent international studies even though differences in study populations and methodology compromise direct comparison. The recent publication from Sweden reported comparable short-term adherence to evolocumab, with 14% having a PDC below 0.8 during the first year of treatment.^26^ In persistence analyses, the authors reported a somewhat higher 1-year drug discontinuation of 24%, but applied a shorter grace period of 56 days.^26^ Similarly, an Italian study including 269 incident users of PCSK9 mAbs found that 20% had a PDC <0.75 whereas 73% were persistent to PCSK9 mAb treatment over a 6-month follow-up period.^28^ Discontinuation was defined liberally with a treatment gap of only 30 days. In contrast, an Italian registry study showed that only 5% had a PDC with PCSK9 mAb treatment below 0.8.^15^ Patients were recruited in centres highly experienced in dyslipidaemia management, which may have resulted in closer follow-up, compared with a nationwide cohort. In a selected cohort of FH patients from Spain, only 4% (*N* = 27) reported discontinuation of PCSK9 mAbs, but data were collected from a telephone survey.^12^

### Clinical implications

Our nationwide cohort study demonstrates significantly lower adherence and persistence to PCSK9 mAbs and oral LLD compared with findings in the landmark lipid trials.^4,5^ The explanation may be less close monitoring in daily clinical practice and a more heterogenic patient population with lower risk perception, higher level of psychological distress, lack of memory, and more self-perceived side effects, all known barriers for adherence to LLDs.^29^ The poor adherence to oral lipid lowering therapies is particularly worrisome, as an opportunity of additive LDL-C effect is missed. Non-adherence to lipid lowering therapy, along with usage of low statin doses, has been proved to be negatively associated with achievement of LDL-C targets in patients with coronary heart disease.^30^

Among ASCVD patients with a history of previous statin discontinuation and/or self-perceived side effects, careful information upon prescription of PCSK9 mAbs and close monitoring during follow-up seems crucial.

Although PCSK9 mAbs reduces LDL-C levels by 50% on top of statin therapy, its negative impact on use of oral LLDs may imply that the net reduction in LDL-C levels on average is significantly lower than in clinical practice. Supporting this, previous studies have demonstrated that patients are less likely to meet LDL-C targets if they are treated onPCSK9 mAbs monotherapy.^31^ Additionally, some patients lack therapeutic response to PCSK9 mAb therapy.^31,32^ If these patients also discontinue oral LLD treatment after initiating PCSK9 mAbs, they are likely without any effective lipid lowering treatment. This should be explored further in model studies or preferably clinical trials supported by health economic analyses.

### Limitations

The completeness and longitudinal follow-up of nationwide individual-level data constitutes the main strengths of this study. PCSK9 mAbs became available in Norway in 2015, and we were thus able to capture all incident PCSK9 mAb users in a nationwide cohort including some clinical characteristics. The Norwegian healthcare system is tax funded with access for all citizens. The use of electronic prescription registries is considered reliable for estimating drug adherence, avoiding recall bias, interviewer bias, and observer bias. Additionally, patients are required to pay a deductible for prescription drugs, which increases the likelihood that they have the intention of taking medication as prescribed.

Some important limitations need to be addressed. First, the PCSK9 mAb treatment indication was based on reimbursement codes which have not been validated in the NDR. However, the risk of misclassification is probably low due to the strict criteria for reimbursement in Norway. Even though only specialists or physicians at hospitals are allowed to prescribe PCSK9 mAb with reimbursement, we cannot exclude the possibility of misclassification of reimbursement codes. Treatment indication was defined by the one reimbursement code registered for the index prescription, and we were therefore unable to identify patients with a combination of FH and ASCVD. Second, we were not able to present the LDL-C levels of the population as nationwide data on lipid profiles do not exist. Nonetheless, we anticipate LDL-C levels at baseline to be above 4.0 (FH) or 5.0 (ASCVD) mmol/L due to strict individual reimbursement rules from 2015 through 2022. Third, we did not have information on reasons for discontinuation of lipid lowering treatment, such as statin intolerance, enrolment in clinical trials, emigration, or palliative care. However, the number of different types of statins dispensed prior to PCSK9 mAb initiation might serve as a proxy for statin intolerance. Fourth, we do not know if the dispensed medicine is consumed. Thus, most likely, adherence and persistence are even worse than reported in the present study. Lastly, the study population mainly includes a high-risk group of Caucasian origin, and the results might not be generalizable to other populations and non-western countries.

## Conclusions

In this nationwide cohort of high-risk incident PCSK9 mAb users, more than one out of two stopped statin treatment while 40% discontinued ezetimibe following initiation of PCSK9 mAbs. We also found that almost one in five patients discontinued treatment with PCSK9 mAbs. The study reveals a major potential for improving persistence and adherence to LLDs among high-risk ASCVD patients, particularly addressing continued statin usage following initiation of a PCSK9 mAb.

## Supplementary Material

qcae099_Supplemental_File

## Data availability

According to Norwegian legislation, the Norwegian Data Protection Authority and the Norwegian Directorate of eHealth, we are not allowed to share original study data publicly.
