# Supplementary material for: Treatment with PCSK9 monoclonal antibodies is associated with discontinuation of oral lipid lowering therapy
Source: Eur Heart J Qual Care Clin Outcomes. 2024 Nov 19;11(8):1290–300. doi: 10.1093/ehjqcco/qcae099 (PMC12714381; doi:10.1093/ehjqcco/qcae099)
Supplement: qcae099_Supplemental_File [file qcae099_supplemental_file.docx]

## **Supplementary files**

Supplementary figure 1: Number of PCSK9 monoclonal antibody users per year

| 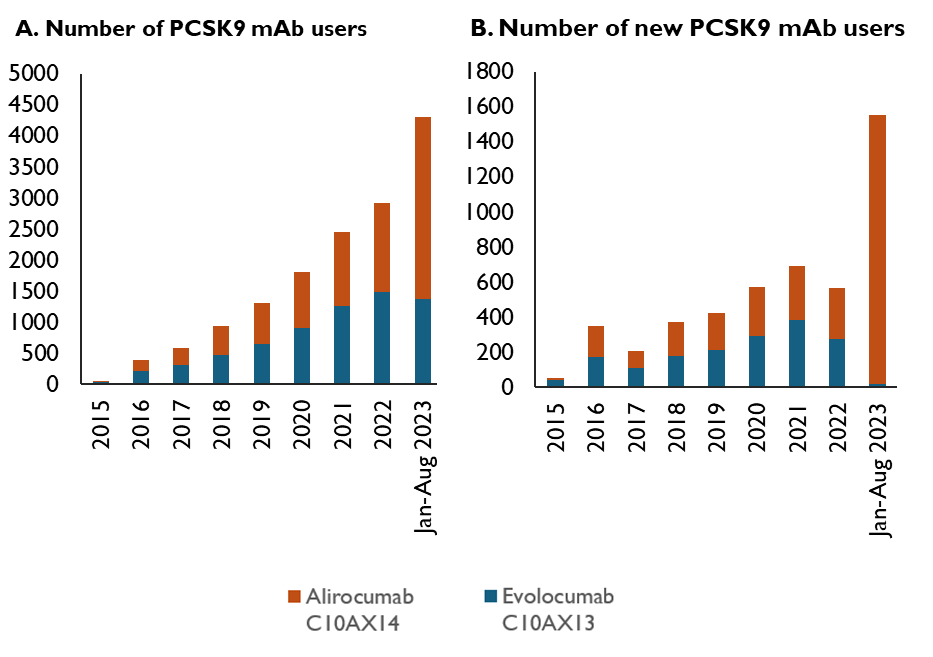 |
| --- |

PCSK9 mAb= Proprotein convertase subtilisin/kexin type 9 monoclonal antibody.

Supplementary table 1: Hazard ratio for PCSK9 mAb discontinuation (180-day treatment gap)

|  | Hazard ratio [95% CI] | P-value |
| --- | --- | --- |
| **Sex** (Reference: male) |  |  |
| Female | 1.63 [1.38- 1.92] | 0.000*** |
| **Statin treatment prior to PCSK9 mAb** (Reference: dispensed <3 statins) |  |  |
| Dispensed ≥3 statins | 1.14 [0.96-1.35] | 0.140 |
| **Age group at index** (Reference: <55) |  |  |
| 55-63 | 0.64 [0.51-0.81] | 0.000*** |
| 64-70 | 0.64 [0.50-0.81] | 0.000*** |
| 71-80 | 0.57 [0.45-0.72] | 0.000*** |
| **Indication** (Reference: ASCVD) |  |  |
| FH | 0.93 [0.76-1.12] | 0.428 |
| Unknown | 2.13 [1.64-2.76] | 0.000*** |
| **Initiation cohort** (Reference: early period initiators) |  |  |
| Late period initiators ^a^ | 0.72 [0.59-0.88] | 0.002** |
| **Region** (Reference: Central) |  |  |
| North | 1.19 [0.83-1.72] | 0.347 |
| South-East | 1.26 [0.95-1.67] | 0.103 |
| West | 1.30 [0.95-1.76] | 0.097 |

^aL^Initiated treatment 2021-august 2022. ASCVD= Atherosclerotic cardiovascular disease; FH= Familial hypercholesterolemia; PCSK9 mAb= Proprotein convertase subtilisin/kexin type 9 monoclonal antibodies. Significance levels: *** <0.001, ** <0.01, * <0.05.

Supplementary figure 2: Persistence to PCSK9 monoclonal antibodies (time until first 90-day treatment gap)

| 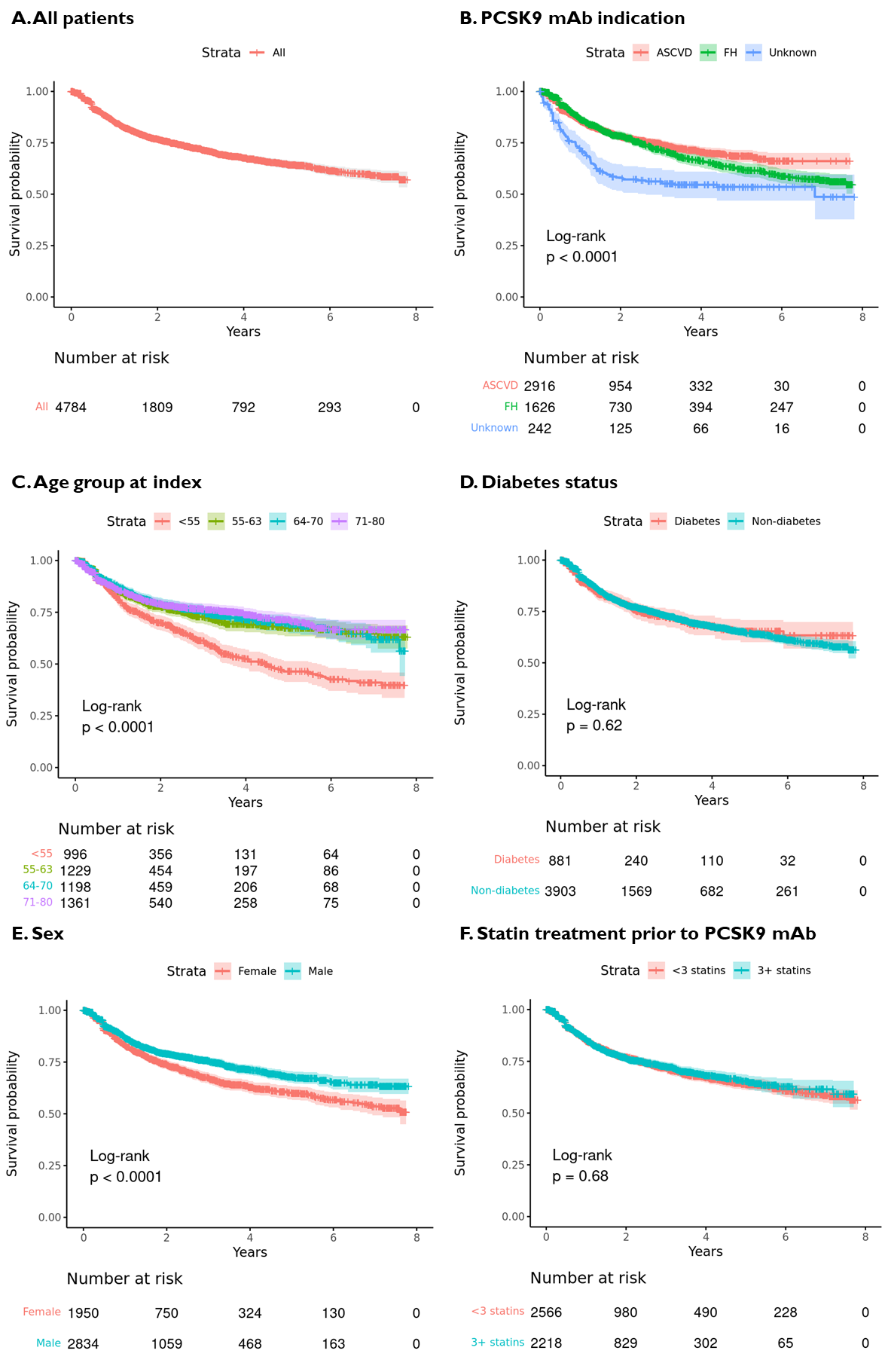 |
| --- |

ASCVD= Atherosclerotic cardiovascular disease; FH= Familial hypercholesterolemia; PCSK9 mAb= Proprotein convertase subtilisin/kexin type 9 monoclonal antibodies. Statin treatment prior to PCSK9 mAb refers to the number of distinct statin types dispensed.
